# Hypoxic-ischemic enterocolitis in extremely low birth weight infants: diagnostic insights from a pilot study

**DOI:** 10.1007/s00431-026-06852-3

**Published:** 2026-04-14

**Authors:** Tainá Cristina Ferrari, Rafael Simone Saia, Cristina Helena Faleiros Ferreira, Karin Barszcz, Walusa Assad Gonçalves-Ferri

**Affiliations:** 1 Department of Pediatrics, Ribeirão, Preto Medical School, University of São Paulo, Bandeirantes Avenue 3900, Ribeirão Preto, São Paulo, Brazil; 2 Department of Physiology, Ribeirão, Preto Medical School, University of São Paulo, Ribeirão Preto, São Paulo, Brazil

**Keywords:** Hypoxic-ischemic enterocolitis, Necrotizing enterocolitis, Preterm infant, Extremely low birth weight

## Abstract

Hypoxic-ischemic enterocolitis (HIEnt) in extremely low birth weight (ELBW) infants represents a significant clinical challenge, frequently occurring in the first week and remaining undetected because of its non-specific presentation. Timely and accurate diagnosis is crucial for improving the poor outcomes associated with this condition. The aim of this pilot study was to evaluate early diagnostic tools and characterize HIEnt in a cohort of ELBW infants during the first week of life, with the goal of identifying promising markers for future large-scale studies. In this prospective, single-center pilot study, 23 ELBW infants (< 1000 g) born from July 2023 to February 2024 were enrolled. Clinical, perinatal, and neonatal variables, along with serum biomarkers (sodium, platelets, bicarbonate, and intestinal fatty acid-binding protein [I-FABP]), were systematically collected on days 2, 4, and 6. HIEnt was diagnosed according to modified Bell’s criteria. Three infants (13%) were diagnosed with HIEnt. HIEnt cases exhibited significantly lower birth weight (mean 568.33 g vs. 818.00 g; *p* = 0.03) and a higher incidence of abnormal antenatal Doppler findings (100% vs. 20%; *p* = 0.02) compared to non-HIEnt infants. Platelet counts were lower in the HIEnt group on day 4 (*p* = 0.06), and initiation of diet was significantly delayed (mean 14.5 days vs. 3.59 days; *p* = 0.04). Serum I-FABP levels did not demonstrate significant differences or consistent temporal trends, and common clinical symptoms did not distinguish between groups.

*Conclusion*: Prenatal risk factors, low birth weight, feeding intolerance, and decreased platelet levels may serve as early indicators of HIEnt in ELBW infants, underscoring the condition’s distinct pathophysiological origins. These findings underscore the need for integrated, multimodal diagnostic strategies and support the pursuit of larger, multicenter validation studies to improve the detection of HIEnt and outcomes in this vulnerable population.

**Table:** 

** What is Known: ** • *Hypoxic-Ischemic Enterocolitis (HIEnt) in extremely low birth weight (ELBW) infants is a distinct and severe condition manifesting within the first week of life. It is frequently underdiagnosed due to non-specific clinical signs and the limited sensitivity of existing diagnostic tools.*	
What is Known: • *This pilot study identifies significant predictors and diagnostic tools, including lower birth weight, abnormal antenatal Doppler findings, early platelet decline, and feeding intolerance, as potential early indicators of HIEnt in ELBW infants during the first week of life, underscoring the need for multimodal diagnostic strategies.*

** What is Known:
**

• *Hypoxic-Ischemic Enterocolitis (HIEnt) in extremely low birth weight (ELBW) infants is a distinct and severe condition manifesting within the first week of life. It is frequently underdiagnosed due to non-specific clinical signs and the limited sensitivity of existing diagnostic tools.*

What is Known:

• *This pilot study identifies significant predictors and diagnostic tools, including lower birth weight, abnormal antenatal Doppler findings, early platelet decline, and feeding intolerance, as potential early indicators of HIEnt in ELBW infants during the first week of life, underscoring the need for multimodal diagnostic strategies.*

## Introduction

Hypoxic-ischemic enterocolitis (HIEnt), also referred to as early necrotizing enterocolitis, is increasingly recognized as a distinct clinical entity in preterm infants, typically presenting within the first week of life [1–3]. The pathogenesis of HIEnt is primarily associated with prenatal hypoxic-ischemic insults, such as a high incidence of small for gestational age (SGA), maternal hypertension, or intrauterine growth restriction (IUGR), which initiate inflammatory responses and intestinal injury soon after birth. In contrast, classic necrotizing enterocolitis (NEC) generally manifests later and is associated with postnatal factors, including feeding practices, bacterial colonization, and inflammatory stimuli [1–3].

Although evidence supporting the hypoxic-ischemic origin of early-onset NEC is increasing, the condition remains underrecognized and is frequently misdiagnosed in clinical practice [4, 5]. HIEnt is often overlooked in the differential diagnosis during the first week of life, with gastrointestinal symptoms attributed to other common neonatal disorders. Such diagnostic delays are associated with rapid clinical deterioration and significantly higher mortality rates in HIEnt [5]. This persistent diagnostic challenge underscores the need to critically evaluate current diagnostic approaches.

Diagnosing HIEnt in extremely low birth weight (ELBW) infants is challenging because of its frequently non-specific clinical presentation [1]. While conventional imaging, especially plain abdominal radiographs, is essential for identifying advanced features such as pneumatosis intestinalis, its sensitivity for detecting early-stage HIEnt or subtle intestinal injury remains limited. This diagnostic limitation highlights the urgent need for more accurate and timely diagnostic modalities [6].

Biomarkers such as platelet counts, sodium, and bicarbonate levels indicate systemic inflammation or physiological disturbances, but they lack the specificity needed for a definitive HIEnt diagnosis [1, 2]. Abdominal ultrasound has attracted interest due to its capacity to provide real-time visualization of bowel alterations, including wall thickness, perfusion patterns, peristalsis, and pneumatosis. This modality may address certain limitations of conventional radiography in detecting early intestinal compromise [7, 8]. Nevertheless, the precise role, standardization, and optimal integration of biomarkers and ultrasound within a comprehensive diagnostic strategy for HIEnt remain subjects of ongoing investigation.

Additional biomarkers, including intestinal fatty acid–binding protein (I-FABP), have shown promise for detecting intestinal mucosal injury before the onset of clinical signs or radiographic changes [9, 10]. I-FABP has been extensively studied for its high sensitivity in detecting enterocyte damage [10]. However, further research is needed to clarify its kinetics and diagnostic accuracy in extremely low birth weight (ELBW) infants with HIEnt.

This pilot study evaluates a multimodal approach evaluating abdominal ultrasound, clinical signs, and multiple biomarkers to detect HIEnt early in critically ill ELBW neonates during the first week of life. The objective is to generate data to inform and justify larger, multicenter investigations, ultimately contributing to improving the HIEnt diagnosis in this vulnerable population.

## Methods

This investigation was a prospective, observational pilot study conducted at the University Hospital from July 2023 to February 2024. The study protocol received institutional ethics committee approval, and prior to enrollment, written informed consent was obtained from the parents or legal guardians of all participating infants. The duration of the data collection period was determined by the predefined study period.

The study was conducted in a highly specialized Neonatal Intensive Care Unit within a quaternary university hospital. This Neonatal Intensive Care Unit (NICU) is classified as Level IV. The neonatology team consists of specialists trained in complex neonatal management and advanced medical procedures.

All preterm infants weighing less than 1000 g (extremely low birth weight, ELBW) born at the University Hospital during the study period were included. Exclusion criteria comprised suspected or confirmed genetic syndromes, congenital heart disease, major or minor physical malformations diagnosed at birth or within the first week, birth outside the institution (outborn), and death before seven days of age.

Diagnosis of hypoxic-ischemic enterocolitis was established using the modified Bell criteria, focusing specifically on stages II and III [11]. These criteria, widely used and the basis for routine neonatal practice in most neonatal units worldwide, were selected to closely replicate real-world scenarios and provide insight into the potential role of biomarkers as adjunctive tools in clinical practice. The non-HIEnt group comprised infants who did not meet the modified Bell criteria.

After HIEnt diagnosis by the modified Bell criteria, an abdominal ultrasound was performed by specialized neonatal staff blinded to laboratory and biomarker data in the HIEnt group. The findings were verified by an expert radiologist to enhance accuracy, following the international guidelines [7, 8]. Infants who did not meet the Bell criteria were not assessed by ultrasound.

Clinical and laboratory data were systematically collected by trained research personnel. Baseline variables included gestational age, birth weight, sex, antenatal corticosteroid use, mode of delivery, and birth weight adequacy (compared with Intergrowth-21st curves).

Maternal and obstetric conditions recorded included chronic hypertension, pre-eclampsia/eclampsia, chorioamnionitis, and abnormal antenatal umbilical artery Doppler results, defined as absent or reversed end-diastolic flow in at least 50% of waveforms or cerebral redistribution. A senior obstetrician with extensive experience performed the maternal ultrasound. Additional birth parameters included the need for resuscitation, 5-min Apgar score, and death outcome.

Serum biomarkers, including I-FABP, platelets, sodium, and bicarbonate, were measured on days 2, 4, and 6 of life. Blood samples (1 mL each) were collected via umbilical catheter at these time points. Samples were placed in gel-containing Vacutainer tubes (BD Biosciences, Curitiba, PR, Brazil), chilled, and transported to the laboratory. Blood was centrifuged at 1200 g for 15 min at 4 °C. Supernatants were aliquoted and stored at –80 °C for later analysis. Biomarkers were measured using ELISA kits according to the manufacturer’s instructions (R&D Systems, Minneapolis, MN, USA).

Infants diagnosed with HIEnt received a standardized treatment protocol under close NICU monitoring. Treatment included immediate cessation of enteral feeds (gut rest), administration of broad-spectrum intravenous antibiotics, and mild therapeutic hypothermia for 48 h. All patients followed the same feeding protocol.

The primary outcomes assessed were the duration of parenteral nutrition (the length of time patients received nutrients intravenously), time to achieve full enteral feeding (defined as tolerating 120 mL/kg/day of nutrients by mouth or tube), and the incidence of surgical intervention for NEC. Secondary outcomes included intestinal failure (defined as removal of at least 40 cm of small intestine with the ileocecal valve or 60 cm without the valve), total length of hospital stay, and death.

Data management included concurrent recording in both paper and electronic case report forms. A secure, access-restricted research database enabled double data entry and periodic audits to ensure completeness and accuracy. All procedures involving biological samples adhered strictly to international biosafety guidelines, including the use of Personal Protective Equipment (PPE), biosafety class II laminar flow hoods, and protocols to prevent laboratory cross-contamination.

The sample size for this pilot study was determined by the study period, with all eligible infants weighing less than 1000 g enrolled. Although the sample size was determined by grant requirements and the study period, it is appropriate for a pilot and hypothesis-generating study, which strengthens the overall study design.

Descriptive statistics included frequencies and percentages for categorical variables. Means or medians with measures of dispersion (standard deviation or interquartile range) were used for continuous variables, stratified by study groups (HIEnt vs. non-HIEnt). Normality of continuous variables was assessed using the Shapiro–Wilk test. Group comparisons were performed using either Student’s *t*-test or the Mann–Whitney *U* test. I-FABP clearance was calculated as the difference between consecutive measurements. Statistical significance was defined as a *p*-value < 0.05.

### Human ethics and consent to participate declarations

The study was conducted in accordance with the ethical standards of the institutional research committee and with the 1964 Helsinki Declaration and its later amendments. The protocol was approved by the Institutional Ethics Committee (CAAE 65578722.2.0000.5440). Written informed consent was obtained from the parents or legal guardians of all participating infants prior to enrollment.

### Funding

**Fig. 1:**
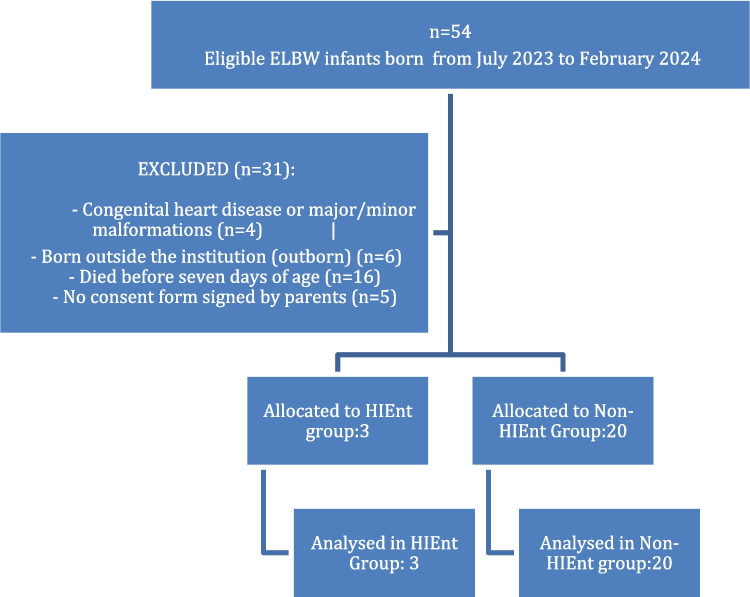
CONSORT flow diagram of study participants

A total of 23 preterm infants were evaluated. During the study period, 54 extremely low birth weight (ELBW) infants were born; four were excluded due to congenital heart disease or major or minor physical malformations diagnosed at birth or within the first week, six were born outside the institution (outborn), sixteen died before seven days of age, and five did not have a consent form signed by parents or guardians (Figure 1).

## Results

A total of 23 preterm infants were evaluated. During the study period, 54 extremely low birth weight (ELBW) infants were born; four were excluded due to congenital heart disease or major or minor physical malformations diagnosed at birth or within the first week, six were born outside the institution (outborn), sixteen died before 7 days of age, and five did not have a consent form signed by parents or guardians (Fig. 1).

The analysis of the neonatal cohort data revealed a mean gestational age of 190.96 ± 11.93 days (approximately 27 weeks and 2 days). The mean birth weight was 785.43 ± 158.21 g, classifying these neonates as extremely low birth weight (ELBW) infants. There were no cases of spontaneous intestinal perforation (SIP) or NEC totalis. The mean onset age was 6.7 days.

Table 1 indicates that birth weight was significantly lower in the HIEnt group (mean 568.33 g, SD ± 60.2) than in the non-HIEnt group (mean 818.00 g, SD ± 146.1; *p* = 0.03). Among the variables compared between HIEnt and non-HIEnt neonates, two demonstrated statistically significant associations. Specifically, altered antenatal Doppler was observed in all HIEnt cases (100%) and in 20% of the non-HIEnt group (*p* = 0.02) (Table 1).

**Table 1:** Comparison of maternal, perinatal, and early clinical characteristics between HIEnt and non-HIEnt preterm infants

Variable	HIEnt (*n* = 3)	Non-HIEnt (*n* = 20)	*p*-value*
Maternal factors			
Systemic arterial hypertension	2 (66.6%)	6 (30%)	0.27
Pre-eclampsia	3 (100%)	7 (35%)	0.07
Eclampsia	0	1 (5%)	1.00
Chorioamnionitis	0	4 (20%)	1.00
Altered Doppler	3 (100%)	4 (20%)	0.02
Cesarean delivery	3 (100%)	11 (55%)	0.25
Neonatal characteristics			
Gestational age (days)	200.00 (± 4.3)	189.60 (± 12.4)	0.11
Birth weight (g)	568.33 (± 60.2)	818.00 (± 146.1)	0.03
Apgar score at 5 min	8.33 (± 0.5)	7.35 (± 2.3)	0.67
Female sex	3 (100%)	8 (40%)	0.09
Small for gestational age (SGA)	3 (100%)	7 (35%)	0.20
Gastrointestinal signs			
Abdominal distension	3 (100%)	11 (55%)	1.00
Vomiting	0	4 (20%)	1.00
Bloody stools	0	0	-
Gastric residuals	3 (100%)	15 (75%)	1.00
Bowel movement during feeding pause	3 (100%)	11 (55%)	1.00

Fisher’s exact test, chi-square test, and Welch’s *t*-test. *HIEnt*, hypoxic-ischemic enterocolitis; *SGA*, small for gestational ege

Other maternal or clinical factors, including systemic arterial hypertension, pre-eclampsia, and small for gestational age, did not show statistical significance. Similarly, classic clinical manifestations such as abdominal distension, gastric residue, and delayed evacuation did not distinguish HIEnt from non-HIEnt infants (Table 1).

Table 2 indicates that the age at diet initiation was significantly higher among HIEnt infants (mean 14.5 days, SD ± 3.5), whereas non-HIEnt infants began feeding substantially earlier (mean 3.59 days, SD ± 2.6; *p* = 0.04). Platelet counts on the fourth day of life were lower in HIEnt infants compared to non-HIEnt infants, but the small sample size limited statistical significance (*p* = 0.06).

**Table 2:** Comparison of key clinical outcomes, feeding parameters, and laboratory values between HIEnt and non-HIEnt preterm infants

Variable	HIEnt (*n* = 3)	Non-HIEnt (*n* = 20)	*p*-value
Duration of parenteral nutrition (days)	42.33 (± 7.7)	21.95 (± 17.7)	0.12
Time to reach 120 mL/kg diet (days)	18.00 (± 25.4)	30.67 (± 16.0)	0.53
Length of hospitalization (days)	73.67 (± 49.1)	142.60 (± 154.2)	0.89
Age at starting diet (days)	14.50 (± 3.5)	3.59 (± 2.6)	0.04
Age at first bowel movement (days)	1.00 (± 0.0)	3.83 (± 3.1)	0.06
Death	2 (66.6%)	6 (30%)	0.53
Chronological age at death (days)	45.5 (7.78)	12.2 (19.4)	0.015
Sodium 2nd day (mmol/L)	134.00 (± 5.2)	137.65 (± 4.4)	0.21
Platelets 2nd day (× 10^3^/µL)	88.00 (± 46.1)	204.50 (± 128.7)	0.11
Bicarbonate 2nd day (mmol/L)	18.00 (± 4.5)	18.15 (± 3.5)	0.96
Sodium 4th day (mmol/L)	133.00 (± 1.7)	135.74 (± 6.9)	0.18
Platelets 4th day (× 10^3^/µL)	54.67 (± 15.5)	155.72 (± 129.9)	0.06
Bicarbonate 4th day (mmol/L)	15.33 (± 5.6)	17.63 (± 3.3)	0.57
Sodium 6th day (mmol/L)	135.00 (± 5.0)	134.50 (± 6.1)	0.78
Platelets 6th day (× 10^3^/µL)	62.00 (± 44.8)	177.69 (± 120.6)	0.10
Bicarbonate 6th day (mmol/L)	17.67 (± 3.0)	17.75 (± 3.5)	0.82

The mean time to achieve full enteral feeds was 18 days (SD ± 25.40) in the HIEnt group and 30.67 days (SD ± 16.0) in the non-HIEnt group, with no statistically significant difference (*p* = 0.53). Although the death rate was not statistically significant between groups (66.6% in HIEnt vs. 30% in non-HIEnt; *p* = 0.53), the chronological age at death was significantly later in the HIEnt group [mean 45.5 days (SD 7.78)] compared to the non-HIEnt group [mean 12.2 days (SD 19.4); *p* = 0.015]. All deaths documented in the study were attributed to clinical sepsis by the clinical staff. No additional variables, including gestational age, Apgar scores, duration of parenteral nutrition, time to achieve full enteral feeding, or laboratory measures such as sodium and bicarbonate, showed statistically significant differences between groups. Neither group required laparotomy.

**Fig. 2:**
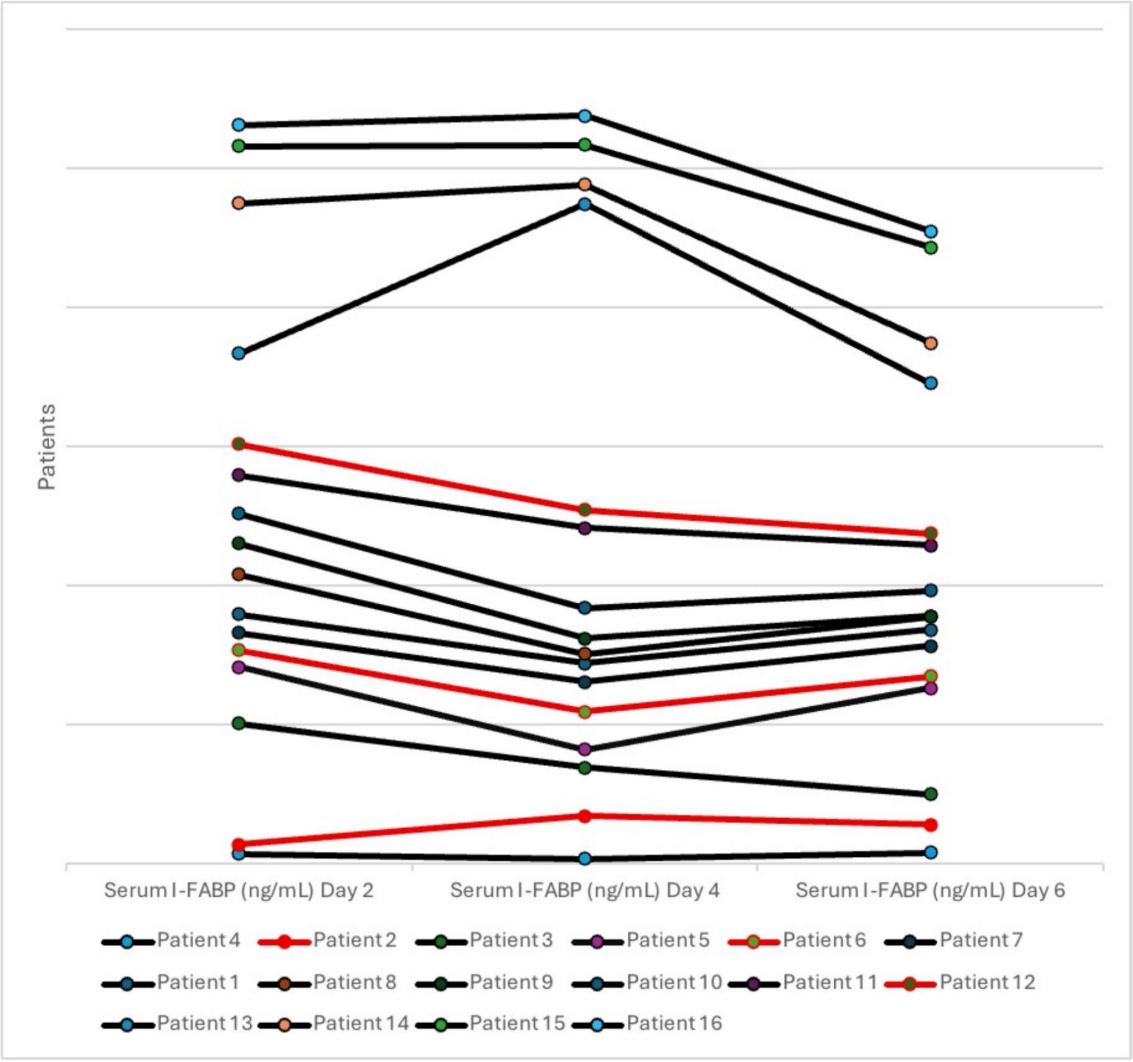
Trends in serum I-FABP (ng/mL) levels for individual patients. The line graph shows the measured serum intestinal fatty acid–binding protein (I-FABP) concentrations, in nanograms per milliliter, for each patient on days 2, 4, and 6. Each colored line corresponds to an individual patient. Patients with incomplete data were excluded from the I-FABP analysis (severe clinical conditions leading to an insufficient blood sample—5 patients). Patients 2, 6, and 12 (indicated by red lines) were classified as hypoxic-ischemic enterocolitis cases. The mean (± standard deviation) serum I-FABP levels were 0.78 ± 0.76 ng/mL on day 2 (*n* = 20), 0.85 ± 1.24 ng/mL on day 4 (*n* = 17), and 0.61 ± 0.52 ng/mL on day 6 (*n* = 13). Analytical comparisons showed no significant differences between day 2 and day 4 (mean paired difference =  + 0.070 ng/mL, *p* = 0.76), day 4 and day 6 (+ 0.021 ng/mL, *p* = 0.92), or day 2 and day 6 (+ 0.049 ng/mL, *p* = 0.84). Clearance values calculated between time points indicated marked individual variability, ranging from − 4.28 to + 2.24 ng/mL; no significant temporal trend or difference was detected (all *p* > 0.05). Results are presented as mean (standard deviation). *p*-values represent group comparisons (Student’s *t*-test or Mann–Whitney *U*). HIEnt, hypoxic-ischemic enterocolitis

Ultrasound examination was conducted exclusively in the HIEnt group. Observed findings included hyperechogenicity of the parietal area, loop hyperemia, and free fluid in two patients (66%). In one patient, only free fluid was detected. Pneumatosis was not observed in the HIEnt group (Fig. 2).

## Discussion

Hypoxic-ischemic necrotizing enterocolitis (HIEnt) remains a diagnostic challenge in the care of critically ill extremely low birth weight (ELBW) neonates [4]. This pilot study sought to identify early clinical, obstetric, and biochemical markers that could improve HIEnt diagnosis in this highly vulnerable population, where the condition is often underrecognized and misdiagnosed due to non-specific symptoms [1, 2].

HIEnt occurred in 13% of cases, with a mean onset at 6.7 days, highlighting the urgent need for early detection. Significant risk factors for HIEnt were identified, enabling improved risk stratification. Infants with HIEnt had lower birth weights (mean 568 g vs. 818 g; *p* = 0.03), and this was associated with maternal vascular compromise. All of these cases involved mothers with abnormal Doppler ultrasound (*p* = 0.02). These results support maternal vascular pathology as a contributor to neonatal intestinal disease [1].

Prolonged fetal hypoxia and ischemia impair intestinal development, leading to mucosal dysfunction, dysmotility, and increased vulnerability to postnatal bacterial invasion. Preterm infants with abnormal antenatal Doppler findings, particularly absent or reversed end-diastolic flow, demonstrate significantly higher rates of necrotizing enterocolitis (HIEnt), feeding intolerance, and delayed achievement of full enteral feeding compared to those with normal Doppler results [12–14].

The risk of HIEnt and other gastrointestinal complications escalates with the severity of Doppler abnormalities, especially when both the umbilical artery and ductus venosus are affected. These Doppler findings independently predict HIEnt risk, irrespective of gestational age or growth restriction, and serve as reliable indicators of hypoxic-ischemic injury and HIEnt in preterm infants. Our data align with existing literature; therefore, these results underscore the importance of proactively monitoring prenatal predictors and implementing an individualized approach to reduce the incidence of HIEnt and related complications [12–14].

Gastrointestinal signs are important for the early detection of enterocolitis in preterm infants; however, these indicators lack sufficient specificity for definitive diagnosis. While existing literature highlights the low diagnostic specificity of clinical signs, the specificity for hypoxic-ischemic enterocolitis has not been thoroughly investigated.

The present study evaluated major clinical symptoms, such as abdominal distension, vomiting, bloody stools, gastric residuals, and alterations in bowel movements during feeding pauses. These findings did not differentiate HIEnt from non-HIEnt infants. Consequently, we reiterate that a multimodal diagnostic approach is warranted. Integrating clinical assessment with advanced imaging techniques and biomarker monitoring is essential to address the diagnostic challenges posed by the non-specific NEC presentation, such as HIEnt [15–17].

Regarding the feasibility of the multimodal approach, biomarkers that require urine and stool samples were not feasible for diagnosis in the first week of life due to non-evacuation and difficulties in collecting urine samples through collectors in ELBW preterm infants. Therefore, laboratory biomarkers and ultrasound remain the most feasible tools for HIEnt diagnosis [18, 19].

Platelet levels measured at 4 days revealed a trend toward significant differences between groups. The HIEnt group had lower platelet counts [54.67 (± 15.5) vs. 155.72 (± 129.9), *p*-value = 0.06]. The literature notes that platelet count and related indices are valuable, accessible, and cost-effective tools for early diagnosis, risk stratification, and prognosis of hypoxic-ischemic necrotizing enterocolitis. A rapid decline in platelet count, especially to levels below 100 × 10^9^/L, is strongly linked to severe disease, bowel necrosis, and the need for surgical intervention. Platelet count can serve as an early biomarker for HIEnt, with 69–83% sensitivity and 83–93% specificity, especially when combined with other markers. Thrombocytopenia occurs in 50–95% of preterm infants with HIEnt and typically develops within 24–72 h of onset. In this cohort, a marginally significant difference in platelet counts was observed at 4 days, with a mean NEC onset of 6.7 days. This difference may have come before clinical diagnosis and showed a pattern distinct from that reported in the literature [20–22].

Table 2 indicates that the age at diet initiation was significantly higher in the HIEnt group. All patients adhered to the same feeding protocol, and fasting was implemented in cases of abdominal distension, pain, or clinical instability. These findings suggest an association between feeding intolerance and HIEnt in this cohort. Although feeding intolerance is not highly specific, it is considered a potential predictor of severe necrotizing enterocolitis (NEC) and remains a key component of NEC severity assessment tools, being included in all NEC scoring systems. Results from this pilot study indicate that this indicator may contribute to a multimodal HIEnt diagnosis [23–25].

The mortality rate was higher in the HIEnt group (66.7%, 2 of 3 patients) than in the non-HIEnt group (30%, 6 of 20 patients), although the difference was not statistically significant (*p* = 0.53). Patients in the HIEnt group died at a significantly later chronological age, with a mean of 45.5 days (SD ± 7.78), compared to 12.2 days (SD ± 19.4) in the non-HIEnt group (*p* = 0.015).

All deaths in this study were attributed to clinical sepsis by the clinical staff. The observed differences in timing of death suggest distinct clinical trajectories. Delayed mortality in the HIEnt group may reflect prolonged illness, chronic complications, or increased vulnerability following the HIEnt episode, rather than an acute, direct HIEnt-related cause. In contrast, earlier deaths in the non-HIEnt cohort may be attributable to immediate complications of extreme prematurity. The death rate associated with enterocolitis Bell stage II was higher than in the pilot study; however, due to study design and sample size, the impact of the proposed NEC treatment (conventional treatment combined with mild hypothermia for 48 h) on mortality could not be directly assessed in this cohort [4, 26, 27].

Serum I-FABP levels did not differ significantly over time or between groups, and no consistent temporal trend was observed (all *p*-values > 0.05). The distinct pathophysiology of HIEnt, which is primarily associated with perinatal hypoxic-ischemic insults, may result in different patterns and kinetics of I-FABP release compared to those observed in classic NEC, where inflammatory and bacterial factors predominate. The pilot study design limited the ability to detect a significant association with serum I-FABP. Larger multicenter studies with optimized collection strategies are necessary to reevaluate the role of I-FABP in HIEnt [9, 10].

Bowel ultrasound (BUS) is a highly accurate, non-invasive modality for diagnosing enterocolitis (NEC), with superior sensitivity and specificity compared to X-ray. However, BUS remains a relatively recent technique and faces several barriers to widespread adoption, including insufficient education and training for sonographers and radiologists, low case volume, and limited clinician familiarity with BUS interpretation. In the present pragmatic study, only the HIEnt group underwent ultrasound assessment due to technical limitations and constraints in study design. This restricted application may limit the generalizability of our findings. Furthermore, our findings differ from those of classic NEC as described in the literature. Consequently, the diagnostic accuracy of BUS for HIEnt warrants further investigation [28–31].

A primary limitation of this pilot study is the small sample size, especially the limited number of patients with hypoxic-ischemic enterocolitis. This constraint reduces the statistical power of the analyses and prevents definitive conclusions about the observed associations. Therefore, the findings should be interpreted with caution and considered as hypothesis-generating rather than conclusive. Although some associations achieved statistical significance, these results require validation in larger, multicenter studies to establish their generalizability and clinical relevance before informing changes in clinical practice or diagnostic algorithms.

A significant limitation of this pilot study is the exclusion of a substantial proportion of eligible extremely low birth weight (ELBW) infants who died before 7 days of age (16 out of 54, representing 29.6% of the initial cohort). The systematic removal of the most severely ill infants, potentially including those with severe early-onset hypoxic-ischemic enterocolitis (HIEnt), introduces a survivorship bias. As a result, the reported incidence and severity profile of HIEnt, as well as the observed biomarker trajectories, may be underestimated or distorted because the most critical cases were excluded from the final analysis. This bias should be considered when interpreting the findings and underscores the challenge of accurately assessing the true burden and earliest indicators of HIEnt in this vulnerable population.

Another significant limitation of this pilot study is the exclusive use of bowel ultrasound (BUS) in infants diagnosed with HIEnt. Although this approach yielded valuable insights into imaging characteristics within the affected group, the lack of BUS data from the non-HIEnt cohort limits the assessment of specificity and false-positive rates for findings such as hyperechogenicity, hyperemia, and free fluid. This methodological constraint, resulting from technical challenges and the pilot design, restricts the interpretability and generalizability of the BUS observations. Therefore, the diagnostic utility of BUS for early HIEnt detection cannot be fully established based on these results, highlighting the need for future large-scale, multicenter studies that systematically include ultrasound assessments for all enrolled infants, regardless of HIEnt status.

This pilot study provides preliminary insights into the diagnosis of hypoxic-ischemic enterocolitis (HIEnt) in extremely low birth weight infants. The development and evaluation of multimodal diagnostic tools that integrate clinical assessment, prenatal predictors, and biomarkers are recommended. Further large-scale multicenter studies are necessary to validate early diagnostic markers and improve strategies for this high-risk population.

## Conclusion

This pilot study investigates the diagnostic challenges associated with hypoxic-ischemic enterocolitis (HIEnt) in extremely low birth weight neonates, which arise due to its non-specific clinical presentation. Preliminary associations have been identified between HIEnt and prenatal risk factors, such as lower birth weight and abnormal antenatal Doppler findings, as well as trends in platelet counts and feeding intolerance. These results suggest that a multimodal diagnostic approach incorporating these markers may be valuable. Further large-scale, multicenter studies are necessary to validate these findings and guide the development of risk assessment tools for HIEnt.

## Data Availability

The data that support the findings of this study are available from the corresponding author upon reasonable request.
